# Mixture Effects of Per- and Polyfluoroalkyl Substances on Embryonic and Larval Sheepshead Minnows (*Cyprinodon variegatus*)

**DOI:** 10.3390/toxics12010091

**Published:** 2024-01-20

**Authors:** Philip Tanabe, Peter B. Key, Katy W. Chung, Emily C. Pisarski, Jessica L. Reiner, Alix E. Rodowa, Jason T. Magnuson, Marie E. DeLorenzo

**Affiliations:** 1 National Oceanic and Atmospheric Administration, National Ocean Service, National Centers for Coastal Ocean Science, Charleston, SC 29412, USA; pete.key@noaa.gov (P.B.K.); katy.chung@noaa.gov (K.W.C.); emily.pisarski@noaa.gov (E.C.P.); marie.delorenzo@noaa.gov (M.E.D.); 2 National Institute of Standards and Technology, Charleston, SC 29412, USA; jessica.reiner@nist.gov; 3 National Institute of Standards and Technology, Gaithersburg, MD 20899, USA; alix.rodowa@nist.gov; 4 U.S. Geological Survey, Columbia Environmental Research Center, Columbia, MO 65201, USA; jmagnuson@usgs.gov

**Keywords:** PFAS, PFOS, PFOA, mixtures, mechanism of toxicity, PPARα, oxidative stress

## Abstract

Per- and polyfluoroalkyl substances (PFAS) are ubiquitous and persistent environmental contaminants originating from many everyday products. Perfluorooctane sulfonic acid (PFOS) and perfluorooctanoic acid (PFOA) are two PFAS that are commonly found at high concentrations in aquatic environments. Both chemicals have previously been shown to be toxic to fish, as well as having complex and largely uncharacterized mixture effects. However, limited information is available on marine and estuarine species. In this study, embryonic and larval sheepshead minnows (*Cyprinodon variegatus*) were exposed to several PFAS mixtures to assess lethal and sublethal effects. PFOS alone was acutely toxic to larvae, with a 96 h LC_50_ of 1.97 mg/L (1.64–2.16). PFOS + PFOA resulted in a larval LC_50_ of 3.10 (2.62–3.79) mg/L, suggesting an antagonistic effect. These observations were supported by significant reductions in malondialdehyde (105% ± 3.25) and increases in reduced glutathione concentrations (43.8% ± 1.78) in PFOS + PFOA exposures compared to PFOS-only treatments, indicating reduced oxidative stress. While PFOA reduced PFOS-induced mortality (97.0% ± 3.03), perfluorohexanoic acid (PFHxA) and perfluorobutanoic acid (PFBA) did not. PFOS alone did not affect expression of peroxisome proliferator-activated receptor alpha (*pparα*) but significantly upregulated apolipoprotein A4 (*apoa4*) (112.4% ± 17.8), a downstream product of *pparα*, while none of the other individually tested PFAS affected *apoa4* expression. These findings suggest that there are antagonistic interactions between PFOA and PFOS that may reduce mixture toxicity in larval sheepshead minnows through reduced oxidative stress. Elucidating mechanisms of toxicity and interactions between PFAS will aid environmental regulation and management of these ubiquitous pollutants.

## 1. Introduction

Per- and polyfluoroalkyl substances (PFAS) are a ubiquitous class of contaminants commonly found in firefighting foams, cookware, clothing, and many other sources [1,2,3]. Due to their amphiphobic properties and chemically inert carbon–fluorine bonds, PFAS have long environmental half-lives and are considered “forever chemicals” [4]. Their persistence and toxicity resulted in some PFAS being listed in the Stockholm Convention as potential human and ecosystem hazards [5]. PFAS have been shown to cause endocrine disruption, reproductive and developmental toxicity, disruptions in lipid homeostasis, and oxidative stress in mammals and fish [6,7]. Despite production bans, PFAS, particularly perfluorooctane sulfonic acid (PFOS) and perfluorooctanoic acid (PFOA), are still ubiquitously found in most environments around the world [8,9,10]. Aqueous film-forming foams (AFFFs), which have been shown to be toxic to several aquatic species [11], are a major source of PFAS pollution in the environment, with a significant proportion coming from legacy and ongoing use on military bases and aircraft carriers [12,13]. PFOS concentrations as high as 8.97 mg/L have been recorded in surface waters near U.S. Air Force bases [14], which far exceeds the US EPA saltwater benchmark for PFOS of 0.55 mg/L [15]. PFAS have also been shown to bioaccumulate within individual organisms and biomagnify across trophic levels [16,17,18].

Although many groups have studied the effects of PFAS on aquatic organisms, over 95% of PFAS fish studies on the US EPA ECOTOX Knowledgebase have been conducted in freshwater species [19]. Salinity can influence PFAS kinetics [20,21,22,23], as well as toxicity [24]. As the ocean serves as a tertiary sink for many PFAS, it is imperative to better understand their effects on saltwater species.

The mechanisms of PFAS toxicity are still largely unknown and seem to differ across individual PFAS. Peroxisome proliferator-activated receptor alpha (PPARα) is a nuclear receptor that regulates lipid and fatty acid metabolism and has been linked to PFAS toxicity [25,26]. There is evidence of PPARα activation by PFAS, particularly those with carboxylic acids, being linked to toxic effects [27]. In contrast, PFAS with sulfonic acids are typically less potent PPAR agonists but induce greater acute toxicity [25]. A potential mechanism of toxicity for sulfonic acids (e.g., PFOS) is the induction of reactive oxygen species (ROS) which results in oxidative stress [28,29,30]. While PPARα activation and ROS generation are potential modes of action for some PFAS species, further work is required to validate these mechanisms.

PFAS are almost always present as mixtures in the environment as a result of their manufacturing processes [1,31], which can affect their kinetics and toxicity. Some compounds can act as agonists of nuclear receptors, such as PPARα, while others can act as antagonists. Furthermore, the expression of the same enzyme can either be upregulated or downregulated depending on the PFAS compound or species [7]. While there is a limited number of PFAS mixture studies conducted in saltwater fish, many other freshwater fish and non-fish species have been used to assess PFAS mixture toxicities, although there is no clear trend towards additive, synergistic, or antagonistic effects [32,33,34,35,36,37,38,39]. To reduce uncertainty in PFAS risk assessments, it is pertinent to better understand the effects of PFAS as mixtures and how selected components can affect toxicity.

In this study, embryonic and larval sheepshead minnows (*Cyprinodon variegatus*) were exposed to PFOS or PFOA, two commonly detected PFAS in aquatic environments, to assess the toxicity of the two compounds individually, as well as in binary mixtures. Larvae were then exposed to PFOS, PFOA, perfluorohexanoic acid (PFHxA), or perfluorobutanoic acid (PFBA) to assess lethal and sublethal effects individually, or as a mixture with PFOS. PFHxA and PFBA were selected because they both have the same functional group (carboxylic acid) as PFOA, differing only by carbon chain length to test the role of carbon chain length in augmenting the toxicity of PFOS. Sublethal endpoints included oxidative stress, quantified by malondialdehyde (MDA) and reduced glutathione (GSH) concentrations, and gene expression of *pparα* and a specific downstream product, apolipoprotein A4 (*apoa4*), quantified by qPCR. The aim of this study was to determine the effects of several PFAS, as individual compounds or mixtures, on sheepshead minnow embryos and larvae and to explore the role of two potential molecular initiating events of PFAS toxicity, *pparα* activation and oxidative stress.

## 2. Materials and Methods

### 2.1. Chemicals

PFOS (CAS: 1763-23-1, 97% purity, Santa Cruz Biotechnology, Dallas, TX, USA), PFOA (CAS: 335-67-1, 95% purity, Millipore Sigma, St. Louis, MO, USA), PFHxA (CAS: 307-24-4, 97% purity, Toronto Research Chemicals, North York, ON, Canada), and PFBA (CAS: 375-22-4, 98% purity, Millipore Sigma, St. Louis, MO, USA) were dissolved in deionized (DI) water and stored at room temperature in a dark environment. Optima-grade methanol (Fisher Chemical, Pittsburgh, PA, USA), HPLC-grade water (Honeywell Burdick and Jackson, Muskegon, MI, USA), and ammonium acetate (Thermo Scientific, Waltham, MA, USA) were used for chromatographic analyses of PFAS in exposure solutions.

### 2.2. Maintenance of Sheepshead Minnow Culture

Adult sheepshead minnows were maintained at a light cycle of 16:8 light/dark in a flow-through system. To collect embryos, adults were randomly collected and transferred to 40 L tanks for spawning at a male/female ratio of 1:2. All fish were fed TetraMin tropical flakes.

### 2.3. Exposure Regime

Embryos were collected, sorted for proper stage and viability, and mixed to avoid batch effects. All embryos were <6 h post-fertilization (hpf). Larvae were hatched from collected embryos and sorted for viability. All larvae were <24 h post-hatch (hph). All exposures were 96 h static-renewal, conducted in glass environmental chambers with test conditions set at 25 °C and a 16:8 light/dark cycle. Embryos were exposed in 20 ppt filtered seawater at a volume of 30 mL in 50 mL glass containers, while larvae were exposed at 200 mL in 230 mL glass containers. All exposures were conducted in triplicate (separate containers) without aeration with a plastic lid to minimize evaporation. Water quality (temperature, dissolved oxygen (DO), salinity, pH) was monitored daily. The following thresholds, based on the American Society for Testing and Materials method E 1241-98 [40], were used to determine acceptable water quality: temperature (25 °C ± 2), DO (>4.00 mg/L), salinity (20 ppt ± 1), and pH (7.80 ± 1.00). Larvae were fed freshly hatched artemia daily ad libitum. Exposure solutions were made fresh daily and renewed 100% every 24 h.

To determine acute toxicity (LC_50_) of PFOS and PFOA, 10 sheepshead minnow embryos and larvae were treated with PFOS (1.25, 2.5, 5, 10 mg/L), PFOA (12.5, 25, 50, 100 mg/L), a mixture of PFOS and PFOA at a 1:1 ratio (1.25, 2.5, 5, 10 mg/L), or 100 mg/L PFOA with PFOS (1.25, 2.5, 5, 10 mg/L). Concentrations were based on the limit of solubility of the tested compounds, which was 10 mg/L for PFOS and 100 mg/L for PFOA, followed by a geometric series to generate lower concentrations. Mortality was assessed after 96 h.

To assess mixture toxicity of PFAS, 20 larvae were treated with 100 mg/L PFOA, PFHxA, or PFBA, with or without 2 mg/L PFOS for 96 h. The PFOS concentration represents the LC_50_ value, while the PFOA concentration represents the no adverse effect concentration (NOAC). Larvae were weighed, rinsed with 20 ppt seawater, flash frozen in liquid nitrogen, and stored at −80 °C for gene expression analysis.

To determine the threshold of the antagonistic effects of PFOA, 20 larvae were treated with 2 mg/L PFOS and co-treated with 6.25, 12.5, 25, 50, or 100 mg/L PFOA for 96 h. Mortality was assessed after 96 h.

To assess the effects of oxidative stress on PFOS + PFOA mixture toxicity, 50 larvae were treated with 2 mg/L PFOS, 12.5 mg/L PFOA, or a binary mixture for 96 h. The PFOA concentration represents the lowest tested concentration that significantly reduced PFOS-induced mortality. Mortality was assessed after 96 h. Larvae were weighed, rinsed with 20 ppt seawater, flash frozen in liquid nitrogen, and stored at −80 °C for oxidative stress assays.

### 2.4. Oxidative Stress Assays

MDA concentrations were quantified in sheepshead minnow larvae whole body homogenate (50 larvae per treatment in triplicate) based on previously published methods [41,42]. To a 1.5 mL centrifuge tube, the sample and 4× volume of cold 50 mM potassium phosphate buffer were added. The tissue was homogenized using a tissue homogenizer on ice for 60 s then centrifuged at 4 °C at 13,000× *g* for 5 min. After centrifuging, 75 µL of the supernatant was transferred to a new 1.5 mL centrifuge tube, to which 10.5 µL of 2% butylated hydroxytoluene was then added. To the tube, 1050 µL of 0.375% thiobarbituric acid was added, then the tube was vortexed. Using a needle, a small hole was poked in the cap of the tube, then the tube was heated at 92 °C for 15 min. After heating, 300 µL supernatant was transferred into a 96 well plate in triplicate to be read at 532 nm on a BioTek Epoch 2 microplate spectrophotometer (Agilent, Santa Clara, CA 95051, USA). MDA concentrations were determined using a standard curve.

GSH concentrations were quantified in sheepshead minnow larvae whole body homogenate (50 larvae per treatment in triplicate) based on previously published methods [43,44,45]. To a 1.5 mL centrifuge tube in an ice bath, the sample and 10× volume of cold 5% sulfosalicylic acid were added. The tissue was homogenized using a tissue homogenizer on ice for 60 s then centrifuged at 4 °C at 13,000× *g* for 5 min. After centrifuging, 100 µL of the supernatant was transferred to a new 1.5 mL centrifuge tube in an ice bath, to which 100 µL cold 5% sulfosalicylic acid was then added. To a new 1.5 mL centrifuge tube, 20 µL supernatant, 478 µL 143 mM sodium phosphate buffer, 114 µL NADPH, 68 µL Ellman’s reagent, and 120 µL DI water were added, then the tube was vortexed. To a 96 well plate, 295 µL of the tube contents was loaded into each well. To each well, 5 µL 50 U/mL glutathione reductase was added, followed by the plate being sealed with an adhesive PCR plate seal, flipped three times to mix, tapped on the benchtop to eliminate bubbles, then read at 412 nm on a BioTek Epoch 2 microplate spectrophotometer every 15 s for 6 time points. GSH concentrations were determined using a standard curve.

### 2.5. Gene Expression Analysis

Gene expression analysis methodologies were based on Magnuson et al. [46]. Pooled larvae (20 larvae per treatment in triplicate) were homogenized with a tissue homogenizer on ice, and total RNA was extracted using a Direct-zol RNA Miniprep Plus Kit (Zymo Research, Irvine, CA 92614, USA). Extracted RNA was analyzed on a NanoDrop spectrophotometer (Thermo Fisher Scientific) to ensure 260/280 ratios were above 1.95. RNA was diluted to a concentration of 1 µg/µL, then 1 µg was reverse transcribed to cDNA using a QuantiTect Reverse Transcription Kit (Qiagen, Germantown, MD, 20874, USA) per the manufacturer’s instructions using an Applied Biosystems Veriti 96 Well Thermal Cycler (Thermo Fisher Scientific, Waltham, MA 02451, USA). Quantitative polymerase chain reaction (qPCR) was performed on an Applied Biosystems 7500 Fast Real-Time PCR System using SsoAdvanced Universal SYBR Green Supermix (Bio-Rad Laboratories, Hercules, CA 94547, USA). Each reaction contained 100 ng cDNA along with a 10 µM concentration of specific forward and reverse primer pairs for targeted genes. Primers were obtained from Integrated DNA Technologies (San Diego, CA, USA), and their efficiencies were calculated before use (Table S1). Thermal cycling conditions for qPCR were as follows for all target genes: denaturation at 95 °C for 5 min, 40 cycles of 10 s denaturation at 95 °C, annealing and extension for 60 s at 54 °C, followed by a melt curve from 54–95 °C at 0.5 °C increments. The housekeeping gene, *β-actin*, was used to normalize gene expression, as there were no significant differences in expression between any treatment and the control. Samples were run in triplicate, and the 2^−ΔΔCt^ method was used to calculate relative fold change [47].

To measure *pparα* activation, the expression of *apoa4*, a sensitive and specific downstream product that is not regulated by another PPAR, was selected based on Nagasawa et al. [48] and was quantified following PFAS treatment.

### 2.6. Water Chemistry Analysis

Water samples were taken at 0 and 24 h of exposure from each treatment and stored at −20 °C in a dark environment until analysis. Due to the lengthy chromatographic method and large volume of solvent used per run, water samples were pooled from each replicate which resulted in one measurement per concentration. All samples were directly injected (10 µL) into an Agilent Infinity II (Agilent, Santa Clara, CA, USA) liquid chromatography instrument attached to a SCIEX Triple Quad 5500+ LC-MS/MS (SCIEX, Framingham, MA, USA). A Zorbax Diol (4.6 mm ID, 12.5 mm, 6 μm particle size) attached to an Agilent InfinityLab Poroshell 120 EC-C18 column (4.6 mm ID, 100 mm, 2.7 μm particle size) was used for separation of PFAS and each sample run with a ramping LC solvent gradient with methanol and nanopure water, each containing 10 mmol/L ammonium acetate. A flow rate of 0.4 mL/min was used, and the column was maintained at room temperature. Two multiple reaction monitoring (MRM) transitions were employed for each PFAS, one for quantitation and the other for confirmation of the PFAS (Table S2). The PFAS water concentrations are available in the Supplementary Materials (Table S3).

### 2.7. Statistical Analyses

Probit analyses were conducted in SAS version 9.4 (SAS Institute, Cary, NC 27513, USA) to calculate LC_50_ concentrations of PFOS, PFOA, and binary mixtures to embryos and larvae. Significant differences between LC_50_ concentrations were determined via a ratio test in SAS version 9.4. All other statistical analyses utilized one-way analysis of variance (ANOVA) with a Tukey HSD post hoc test and were conducted in GraphPad version 9.4.1 (GraphPad Software, Boston, MA 02110, USA, www.graphpad.com (accessed on 9 January 2024)). All data were assessed for normality and homogeneity of variance by plotting residuals and quantiles of the data sets. A *p*-value of 0.05 was utilized for all statistical analyses.

## 3. Results

### 3.1. Acute Toxicity of PFOS and/or PFOA on Embryonic and Larval Sheepshead Minnows

Neither PFOS nor PFOA was acutely toxic to sheepshead minnow embryos at any tested concentration as individuals (Table 1). PFOS was acutely toxic to larvae, with an LC_50_ of 1.97 mg/L (95% confidence interval: 1.64–2.16), while PFOA was not toxic at any tested concentrations. PFOS + PFOA exposed at a 1:1 ratio yielded an LC_50_ of 1.99 mg/L (1.70–2.17) which was not statistically different to PFOS-only LC_50_ (*p* > 0.05). However, PFOS co-treated with a constant 100 mg/L PFOA yielded an LC_50_ of 3.10 (2.62–3.79) which was significantly higher (57.4%) than PFOS alone (*p* < 0.05).

### 3.2. Mixture Toxicity of PFOS with PFOA, PFHxA, or PFBA

**Figure 1:**
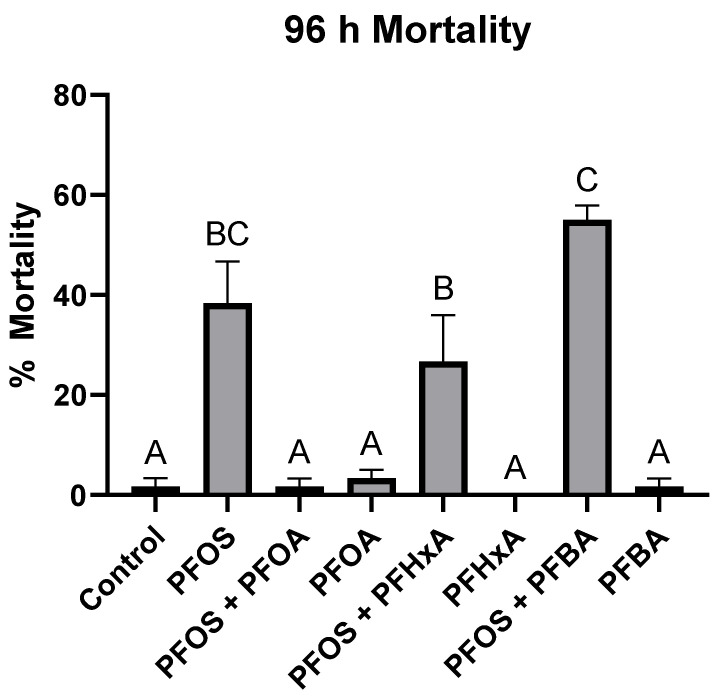
Percent mortality of larval sheepshead minnows (*Cyprinodon variegatus*) after a 96 h exposure to various PFAS mixtures. PFOS = 2 mg/L, and PFOA, PFHxA, and PFBA = 100 mg/L. Letters indicate significant differences determined by a one-way ANOVA followed by a Tukey HSD post hoc test. Values represent the mean of three replicates ± standard error of the mean.

PFOS at 2 mg/L was the only acutely toxic compound when exposed individually, while PFOA, PFHxA, and PFBA at 100 mg/L did not cause significant mortality compared to the control (Figure 1). However, when exposed as a mixture with PFOS, PFOA was the only compound to significantly reduce PFOS-induced mortality (97.0% ± 3.03), while PFHxA and PFBA did not result in statistically different levels of mortality compared to the PFOS-only treatment.

### 3.3. Effects of PFAS on Gene Expression

**Figure 2:**
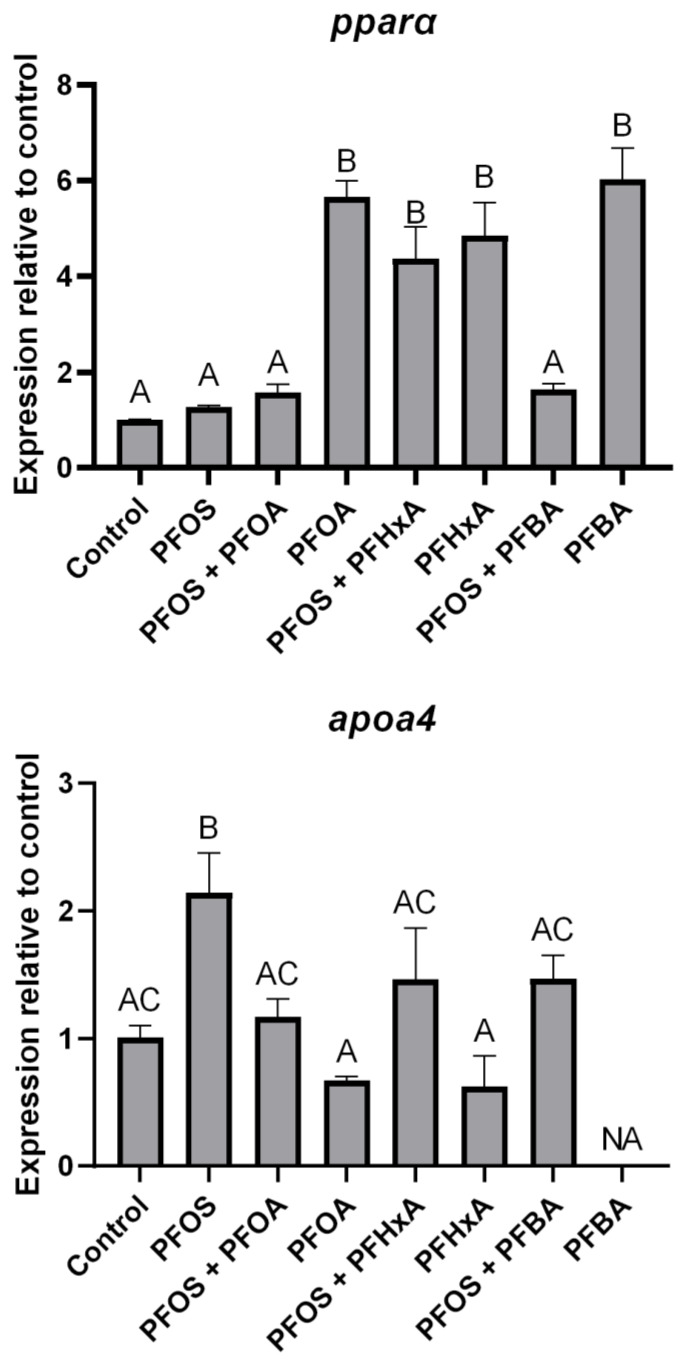
Relative gene expression of *pparα* and *apoa4* following a 96 h exposure of larval sheepshead minnows (*Cyprinodon variegatus*) to various PFAS mixtures. PFOS = 2 mg/L, and PFOA, PFHxA, and PFBA = 100 mg/L. Letters indicate significant differences determined by a one-way ANOVA followed by a Tukey HSD post hoc test. Values represent the mean of three replicates ± standard error of the mean. NA indicates the missing PFBA *apoa4* expression due to technical errors across several replicates.

*Pparα* was significantly upregulated by PFOA (565.4% ± 27.2), PFHxA (482.8% ± 61.4), and PFBA (605.6% ± 51.5) at 100 mg/L compared to the control, while PFOS at 2 mg/L did not statistically affect *pparα* expression (Figure 2). However, PFOS + PFOA and PFOS + PFBA treatments resulted in significant reductions in *pparα* expression compared to treatments without PFOS (72.3% ± 1.64 and 72.4% ± 3.87, respectively). *Apoa4* expression was significantly upregulated following treatment with 2 mg/L PFOS (112.4% ± 17.8) compared to the control. PFOS co-treatment with 100 mg/L PFOA resulted in a significant reduction (45.4% ± 3.78) of *apoa4* expression compared to the PFOS-only treatment. All other treatments did not result in significant changes to *apoa4* expression. Due to technical errors in several replicates, statistical analyses were not feasible for determining *apoa4* expression in the PFBA treatments, so those results were excluded.

### 3.4. Antagonistic Effects of PFOA on PFOS-Induced Mortality

**Figure 3:**
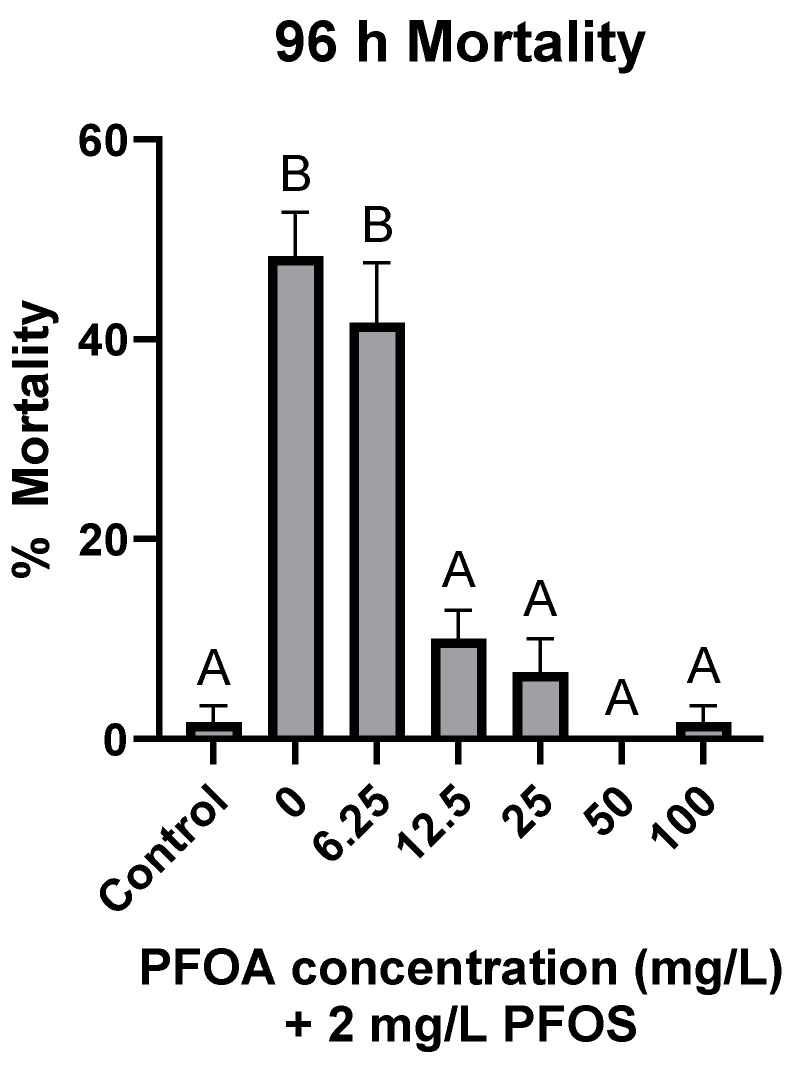
Mortality of larval sheepshead minnows (*Cyprinodon variegatus*) after 96 h exposure to 2 mg/L PFOS with various concentrations of PFOA. PFOS was kept constant at 2 mg/L, while PFOA concentrations ranged from 6.25 to 100 mg/L. Control indicates treatments without PFOS nor PFOA, while 0 indicates 0 mg/L PFOA + 2 mg/L PFOS. Letters indicate significant differences determined by a one-way ANOVA followed by a Tukey HSD post hoc test. Values represent the mean of three replicates ± standard error of the mean.

When co-treated with 2 mg/L PFOS, PFOA significantly reduced PFOS-induced mortality at 12.5, 25, 50, and 100 mg/L, while mortality at 6.25 mg/L did not significantly differ from PFOS-only treatments (Figure 3). At the threshold concentration of 12.5 mg/L, PFOA reduced PFOS-induced mortality by 80.1% ± 4.26.

### 3.5. Effects of PFOS and PFOA on Oxidative Stress

**Figure 4:**
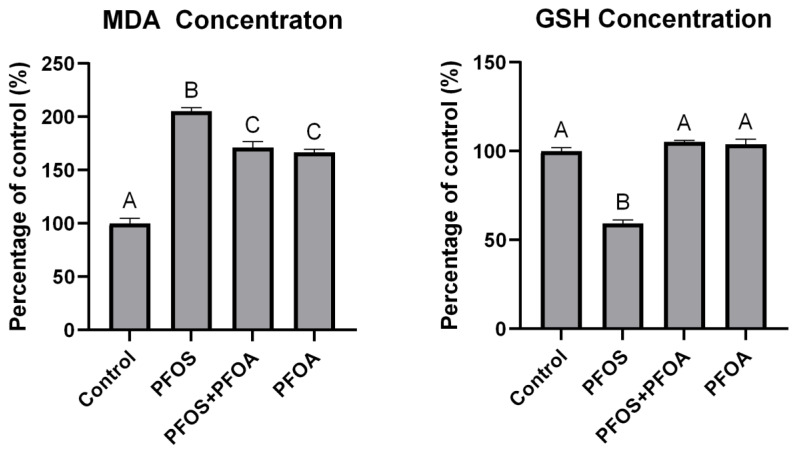
Malondialdehyde (MDA) and reduced glutathione (GSH) concentrations in larval sheepshead minnows (*Cyprinodon variegatus*) exposed to PFOS, PFOA, or a mixture for 96 h. PFOS = 2 mg/L and PFOA = 12.5 mg/L. All concentrations were normalized to the control. Letters indicate significant differences determined by a one-way ANOVA followed by a Tukey HSD post hoc test. Values represent the mean of three replicates ± standard error of the mean.

All treatments significantly increased MDA concentrations relative to the control, with 2 mg/L PFOS increasing MDA by 105% ± 3.25, 2 mg/L PFOS + 12.5 mg/L PFOA by 71.4% ± 5.36, and 12.5 mg/L PFOA by 66.5% ± 3.18 (Figure 4). PFOS + PFOA significantly reduced MDA concentrations compared to PFOS-only treatments by 16.5% ± 2.61. GSH concentrations were significantly reduced by PFOS-only treatments (40.7% ± 1.87), while PFOS + PFOA and PFOA-only treatments did not result in significant changes. GSH concentrations were significantly higher (43.8% ± 1.78) in PFOS + PFOA treatments compared to PFOS only. A PFOA concentration of 12.5 mg/L was used as this was the determined threshold concentration of antagonistic effects with PFOS determined in Figure 3.

## 4. Discussion

Treatments with PFOS or PFOA, as well as binary mixtures to sheepshead minnow embryos did not result in significant mortality. However, larvae treated with PFOS experienced significant mortality, with an LC_50_ of 1.97 mg/L (1.64–2.16), while PFOA treatments did not result in any significant mortality (Table 1). These results are in agreement with Burcham et al. [24] where survival of sheepshead minnow embryos was not affected by PFOS exposure, while larvae were more sensitive. This lack of embryonic sensitivity may result from the chorion preventing the uptake of the chemicals. Similar trends of embryos being resistant to chemical toxicity have been observed with polychlorinated biphenyls (PCBs) [49], 2,3,7,8-tetrachlorodibenzo-*p*-dioxin (TCDD) [50], and bifenthrin [51], all of which are known to be more toxic in larvae. This is a known occurrence mainly for hydrophobic chemicals [52] but can also be influenced by several other factors, such as species-specific chorion properties and exposure conditions [53,54]. PFAS have amphiphobic properties, so it is unclear as to how they interact with the chorion, but these results suggest that the chorion may be hindering PFAS uptake. However, Ankley et al. [55] observed PFOS uptake in embryonic fathead minnows (*Pimephales promelas*). Fang et al. [56] have also reported increased body burdens of PFOS in marine medaka (*Oryzias melastigma*) embryos at 10 days post fertilization (dpf) relative to 4 dpf. Differential gene expression and biomarker concentrations were observed in treated embryos, which indicates PFOS uptake rather than only adsorption to the chorion [56]. Since they have also reported that embryos were less sensitive than larvae, it is possible that embryos may be less sensitive to PFOS than larvae independent of the chorion, but further work on presence or absence of the chorion is warranted to better characterize this difference in life stage-dependent toxicity.

While 1:1 exposures of PFOS + PFOA at all concentrations did not result in significant differences in mortality compared to PFOS only, when PFOA was kept constant at 100 mg/L during co-treatments with PFOS, the LC_50_ was significantly higher at 3.10 mg/L (2.62–3.79), suggesting an antagonistic effect of the mixture (Table 1). PFOS + PFOA co-treatments significantly reduced PFOS-induced mortality even at PFOA concentrations of 12.5 mg/L (Figure 3), while co-treatments of PFOS with PFHxA or PFBA did not significantly affect mortality (Figure 1). However, the level of mortality was significantly different between the three co-treatments. These results indicate that the length of the carbon chain may correlate with the level of observed toxicity, as all three compounds had the same carboxylic acid functional group and only differed by the number of carbons in their backbone. A trend of increased PFAS toxicity has been observed with increased carbon chain length and the presence of a sulfonate group [30,35,57], but to our knowledge, this is the first study which provides evidence of increased antagonistic effects of carboxylic acids with increasing carbon chain lengths co-treated with PFOS. In a similar study using zebrafish (*Danio rerio*) embryos, Ding et al. [32] observed a complex interactive effect of a PFOS-PFOA mixture, observing antagonistic, additive, and synergistic effects depending on the concentration of each compound. Conversely, Menger et al. [35] observed only reduced toxicity in zebrafish embryos exposed to a mixture of nine PFAS compared to individual PFAS. There are few PFAS mixture studies utilizing fish, but even in non-fish models, such as the American bullfrog (*Rana catesbeiana*) [33], Northern leopard frog (*Rana pipiens*) [34], mouse (*Mus musculus*) [36], and Northern bobwhite quail (*Colinus virginianus*) [37,38,39], there are no clear patterns of antagonistic, additive, or synergistic effects of PFAS mixtures.

PPARα activation has been proposed to be a molecular initiating event for toxic effects of many PFAS [58,59]. However, PFOS, which induced the greatest toxic response, did not significantly affect *pparα* expression, while PFOA, PFHxA, and PFBA all significantly upregulated *pparα* while not causing significant mortality (Figure 2 and Figure 3). This was expected as there is a robust weight of evidence toward PFOA having a higher binding affinity toward PPARα in human and rodent cells [27,59,60,61,62]. However, direct binding affinity to PPARα has also been measured in human and Baikal seal (*Pusa sibirica*) cells using competitive binding assays and, in both species, PFOS had a higher binding affinity than PFOA [63]. Several in vivo studies also report conflicting results. Fang et al. [57] observed that PFOS significantly downregulated *pparα* expression in marine medaka embryos at 4 dpf but upregulated it at 10 dpf. Interestingly, Søderstrøm et al. [64] observed synergistic activation of Atlantic cod (*Gadus morhua*) *pparα* when exposed to a PFOS–PFOA mixture compared to PFOA only, as well as to PFOS, which was found to not activate *pparα* on its own. A putative allosteric binding site was identified on the PPARα which, upon binding with PFOS, stabilizes the receptor and potentially increases the binding affinity toward PFOA. However, in the present study, the expression of *pparα* was not affected when sheepshead minnow larvae were treated with PFOS or a PFOS + PFOA mixture but was significantly upregulated when treated with PFOA. Several other studies have also observed upregulation of *pparα* in response to PFOS treatment in zebrafish embryos [29], juvenile Atlantic salmon (*Salmo salar*) [65], and marine medaka [57]. While there is no clear trend for whether PFOS or PFOA have a greater binding affinity toward PPARα in fish, Rosen et al. [26] estimated that over 75% of differentially expressed genes of PFAS-treated mice were PPARα-dependent, indicating the importance of the receptor to PFAS-mediated toxicity.

In contrast to *pparα*, *apoa4* expression was only significantly upregulated in the PFOS treatment, while expression in the PFOS + PFOA co-treatment did not differ from the control or PFOA treatment. While not statistically significant, a trend of increasing *apoa4* expression was also observed in PFOS co-treatments with PFHxA or PFOA which indicates that PFOS may be driving this upregulation. Other groups have observed mixed results with *apoa4* expression following PFAS treatment, as some groups have reported upregulation in PFOA-treated fish [66], while others reported downregulation [67]. While few groups have studied *apoa4* expression in PFOS-treated fish, the same mixed trend was observed in PFOS-treated fish with *apoa1* [29,68]. In rat hepatoma cells, PFOS was found to upregulate *apoa4* roughly two-fold higher than PFOA when exposed at 100 µM but downregulated *apoa4* at 10 µM while PFOA upregulated, and both compounds downregulated *apoa4* at 1 and 0.1 µM [69]. While PFAS have been shown to affect lipid homeostasis and bioenergetics in several species [70,71,72], it is unknown how changes in apolipoprotein concentrations can influence PFAS toxicity, although some groups suggest that they may act as a transporter of PFAS throughout the body [73], and their concentrations are often positively correlated to PFOS and PFOA concentrations in humans [74]. It should also be noted that while *apoa4* was used as a molecular biomarker to confirm *pparα* activation, the inverse trend was observed where PFOS did not upregulate *pparα* while upregulating *apoa4*, while PFOA upregulated *pparα* but not *apoa4* (Figure 2). It is unclear as to why this inversed trend was observed, but differences in the concentrations of PFOS and PFOA may have contributed to this observation. Since PFOS exposures were conducted at 2 mg/L and PFOA at a higher concentration of 100 mg/L, *pparα* regulation may have been affected differently, but the lack of *pparα* upregulation in the PFOS + PFOA co-treatment contrasts this possibility. The difference in *apoa4* concentration, despite the differences in *pparα* regulation, could be due to differences in receptor activation. While *pparα* was shown to be upregulated by PFOA, it does not necessarily mean the receptors were being activated. Conversely, PFOS could also have been activating *pparα* but not upregulating its gene expression. While there exist standardized fluorometric assays for other receptors (e.g., ethoxyresorufin-O-deethylase assay for the aryl hydrocarbon receptor), it is difficult to confirm PPARα activation without specialized means and was outside of the scope of this work. Lastly, while APOA4 is not regulated by other PPARs, it can be regulated by liver X receptors (LXR) in mice [75] and zebrafish [76]. This indicates that a more specific, targeted gene should be used for confirming PPARα activation in future studies.

Oxidative stress through ROS generation has been proposed as a potential mechanism of toxicity of PFAS. Sheepshead minnow larvae treated with 2 mg/L PFOS and 12.5 mg/L PFOA were found to have significantly lower MDA concentrations compared to PFOS-only treatments, indicating lower lipid peroxidation in the mixture compared to the individual PFOS treatment (Figure 4). PFOS also significantly lowered GSH concentrations compared to the control, while PFOS + PFOA co-treatment did not result in significant reductions. This provides evidence toward reduced oxidative stress potentially playing a role in the reduced toxicity of PFOS + PFOA mixtures compared to PFOS-only treatments. β-oxidation of fatty acids, which is regulated by PPARα [77], has been proposed as a potential mechanism of ROS generation via PFAS [7]. However, the oxidative stress and toxicity results did not positively correlate with *pparα* expression in the present study. It is unknown if this is due to differences in life stages, if the mechanisms are species-specific, or a different reason. While PFOS and other sulfonic acid-containing PFAS have been attributed as major contributors to PFAS-induced oxidative stress [29,30,78], both PFOS and PFOA have been shown to up- or downregulate several oxidative stress genes in myriad fish species (summarized in Lee et al. [7]). An ROS-generating mechanism of PFOS has been explored in zebrafish (Shi and Zhou [28]), but there are few comparisons between other individual PFAS and mixtures, warranting further study.

## 5. Conclusions

In summary, none of the tested PFAS caused mortality in embryonic sheepshead minnows at the tested concentrations, while only PFOS caused mortality in larvae. These results suggest that sheepshead minnow embryos are not sensitive to PFAS. Co-treatment with PFOA significantly reduced mortality, as well as lipid peroxidation and depletion of glutathione in PFOS-treated larvae. PFOS was the only compound not capable of upregulating *pparα* expression but was the only compound to upregulate *apoa4* expression in larvae. These findings present preliminary evidence for antagonistic interactions between PFOA and PFOS, which may contribute to reduced mixture toxicity in larval sheepshead minnows, potentially through reduced oxidative stress. Further studies are needed to determine if similar results are observed at environmentally relevant concentrations. Further mechanistic studies are also warranted to explore these antagonistic effects and to identify molecular initiating events to better predict the toxic effects of PFAS mixtures in developing fish.

## Figures and Tables

**Table 1:** Embryonic and larval LC_50_ values for sheepshead minnows (*Cyprinodon variegatus*) exposed to PFOS or PFOA. Values in parentheses indicate 95% confidence intervals. Asterisk (*) indicates significant differences compared to PFOS-only LC_50_ determined by an LC_50_ ratio test.

Chemical	Embryonic LC_50_ (mg/L)	Larval LC_50_ (mg/L)
PFOS	>10	1.97 (1.64–2.16)
PFOA	>100	>100
PFOS + PFOA (1:1)	>10	1.99 (1.70–2.17)
PFOS + 100 mg/L PFOA	>10	3.10 (2.62–3.79) *

## Data Availability

Data is available upon request.

## Supplementary Materials

The following supporting information can be downloaded at: https://www.mdpi.com/article/10.3390/toxics12010091/s1. There are three supplementary tables and one supplementary figure. Table S1, primer sequences used for qPCR. Table S2, instrumental conditions for LC/MS analyses of PFAS. Table S3, measured PFAS concentrations in exposure solutions after 0 or 24 h exposure. Figure S1, relative gene expression of *pparα* and *apoa4* consolidated into one figure.
