# Supplementary material for: Mixture Effects of Per- and Polyfluoroalkyl Substances on Embryonic and Larval Sheepshead Minnows (Cyprinodon variegatus)
Source: Toxics. 2024 Jan 20;12(1):91. doi: 10.3390/toxics12010091 (PMC10819493; doi:10.3390/toxics12010091)
Supplement: Supplementary file 1 [file toxics-12-00091-s001.zip › toxics-2810471-supplementary.pdf]

Supplementary Materials

Mixture effects of PFAS on embryonic and larval sheepshead minnows (*Cyprinodon variegatus*)

Philip Tanabe[1], Peter Key[1], Katy Chung[1], Emily Pisarski[1], Jessica Reiner[2], Alix Rodowa[3], Jason Magnuson[4], Marie DeLorenzo[1]*

[1]National Oceanic and Atmospheric Administration, National Ocean Service, National Centers for Coastal Ocean Science, Charleston, SC

[2]National Institute of Standards and Technology, Charleston, SC

[3]National Institute of Standards and Technology, Gaithersburg, MD

[4]U.S. Geological Survey, Columbia Environmental Research Center, Columbia, MO

There are 5 pages with 1 Supplementary Figure and 3 Supplementary Tables.

** Corresponding author:*

Corresponding author: Philip Tanabe

Corresponding email: philip.tanabe@noaa.gov

Corresponding address: Hollings Marine Laboratory, National Oceanic and Atmospheric Administration, 331 Fort Johnson Rd, Charleston, SC 29412

Table S1. Primer sequences used for qPCR. All forward primers are 5’ to 3’ and reverse primers are 3’ to 5’.

Gene	Direction	Sequence	Amplicon	Accession number
β-actin	Forward	AACACTGTGCTGTCTGGAGGTA	219	XM_015378596.1
	Reverse	AGACGGAGTATTTACGCTCTGG		
pparα	Forward	GACCATCCGTCTGAAGCTGGAGTAT	100	XM_015389442.1
	Reverse	GCACTTCTGGAAGCGGCAGTATT		
apoa4	Forward	AGACTCAGGCTGCTCCTCTCA	99	XM_015391647.1
	Reverse	CCAAAGCGGTCAAATCGTTCTCCA		

Table S2. PFAS analytes, acronyms, diagnostic ions used in the determination of the targeted analytes, depolarization energy (DP), Entrance Potential (EP), collision energy for the product and qualifier ions (CE M_2, CE M_3), and Collision Cell Exit Potential (CXP).

**Analyte**	**Acronym**	**Selected diagnostic ions *m/z***			**DP**	**EP**	**CE M_2**	**EXP**	**CE M_3**
		**Precursor M_1**	**Product M_2**	**Qualifier M_3**					
Perfluoro-n-butanoic acid	PFBA	213	169	N/A	-70	-10	-20	-16	N/A
Perfluoro-n-hexanoic acid	PFHxA	313	269	119	-70	-10	-10	-16	-10
Perfluoro-n-octanoic acid	PFOA	413	369	169	-70	-10	-20	-16	-30
Perfluoro-1-octanesulfonate	PFOS	499	80	99	-60	-10	-60	-16	-60
Perfluoro-n-[$^{13}C_4$]-butanoic acid	13C4-PFBA	217	172	N/A	-70	-10	-20	-16	N/A
Perfluoro-n-[1,2,3,4,6-$^{13}C_5$]-hexanoic acid	13C5-PFHxA	318	273	N/A	-70	-10	-10	-16	N/A
Perfluoro-n-[$^{13}C_8$]-octanoic acid	13C8-PFOA	421	376	N/A	-70	-10	-20	-16	N/A
Perfluoro-1-[1,2,3-$^{13}C_3$]-hexanesulfonate	13C8-PFOS	507	99	N/A	-60	-10	-60	-16	N/A

Table S3. PFAS concentrations in exposure solutions measured at t=0 and t=24. Percent (%) nominal represents measured concentrations relative to nominal concentrations as a percentage after 0 or 24 h exposure.

Chemical	Nominal Concentration (mg/L)	Concentration t=0 (mg/L)	% Nominal t=0	Concentration t=24 (mg/L)	% Nominal t=24
PFOS	1.25	1.42	113	1.22	97
	2	1.71	86	1.42	71
	2.5	1.91	76	2.14	85
	5	3.98	80	3.38	68
	10	6.40	64	7.03	70
PFOA	6.25	6.90	110	7.82	125
	12.5	14.09	113	13.8	110
	25	25.55	102	21.21	85
	50	46.81	94	46.86	94
	100	97.87	98	88.55	89
PFHxA	100	128.2	128	111.8	112
PFBA	100	126.7	127	146.9	147

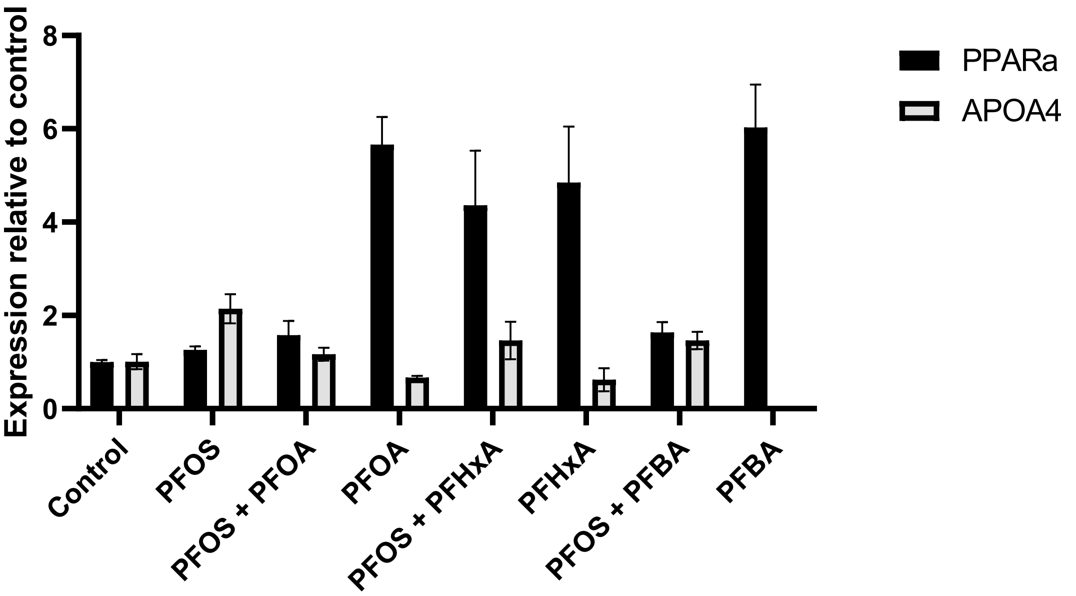

Figure S1. Relative gene expression of *ppara* and *apoa4* following 96 h exposure of larval sheepshead minnows to various PFAS mixtures. PFOS = 2 mg/L and PFOA, PFHxA, and PFBA = 100 mg/L. Values represent the mean of three replicates ± SEM.
